# Cadmium-induced oxidative stress in Prussian carp (*Carassius gibelio* Bloch) hepatopancreas: ameliorating effect of melatonin

**DOI:** 10.1007/s11356-019-04595-3

**Published:** 2019-03-05

**Authors:** Ewa Drąg-Kozak, Dorota Pawlica-Gosiewska, Katarzyna Gawlik, Magdalena Socha, Grzegorz Gosiewski, Ewa Łuszczek-Trojnar, Bogdan Solnica, Włodzimierz Popek

**Affiliations:** 1 Department of Ichthyobiology and Fisheries, University of Agriculture in Krakow, ul. Spiczakowa 6,, 30-199 Krakow-Mydlniki, Poland; 2 Department of Clinical Biochemistry, Jagiellonian University Medical College, Krakow, Poland

**Keywords:** Cadmium, Melatonin, Oxidative stress, Prussian carp, Bioaccumulation, Depuration

## Abstract

The oxidative status of the hepatopancreas of Prussian carp females (*Carassius gibelio*) co-exposed to sublethal cadmium in water and melatonin was studied. The activities of antioxidant enzymes such as glutathione reductase (GR), glutathione peroxidase (GPx), and superoxide dismutase (SOD) as well as the concentration of reduced glutathione (GSH) were measured in homogenates of the hepatopancreas. Furthermore, concentrations of cadmium (Cd), zinc (Zn), copper (Cu), and iron (Fe) in the hepatopancreas were assayed. These females received melatonin implants and were exposed to 0.4 mg/L or 4.0 mg/L Cd in water for either a 13- or a 7-week period, followed by further 6 weeks of purification in clear water. Exposure to Cd influenced the increase in this metal concentration in fish hepatopancreas. In contrast, the fish exposed to cadmium with additional administration of melatonin had a lower accumulation of this metal. Exposure to Cd caused the increase in GSH content and the activity of GR, and a reduction in GPx activity, whereas the SOD activity varies depending on the exposure time on cadmium. In the hepatopancreas of fish treated with Cd alone, the content of Cu and Zn were increased and that of Fe was changed. After melatonin administration to Cd-exposed fish, a decrease in copper and zinc hepatopancreas content was noted. The present findings imply that melatonin co-treatment can effectively protect the fish against the toxic effects of cadmium on endogenous antioxidant status in hepatopancreas tissues and variations in metal concentration, such as Zn, Cu, and Fe.

## Introduction

Human activity and civilization causes increasing heavy metal pollution. This is particularly evident in inland groundwater. Heavy metals such as lead, mercury, zinc, or cadmium infiltrate the water environment with surface runoff or industrial and agricultural wastewater. Heavy metals are not biodegradable but are easily absorbed and accumulated both in the water environment and in organisms that inhabit it.

Cadmium is considered a major chemical pollutant of the aqueous environment and a serious threat to water organisms, especially fish (Derective 2013). Environmental exposure to cadmium may lead to the absorption of large quantities of the element, causing harm to the organism (Abalaka 2015).

Studies on various species of fish revealed that cadmium may affect physiological and biochemical processes in tissues and organs (Al-Asgah et al. 2015). This metal accumulates in different organs (liver, pancreatic gland, kidneys, intestine, gills etc.) causing histopathological changes in them (Abalaka 2015). Cadmium toxicity is conditioned by many different mechanisms. The results obtained in studies conducted in vitro and in vivo show that cadmium takes part in the generation of reactive oxygen species (ROS) and their derivatives. The effects of Cd administrated in high concentrations have been attributed to an excessive increase in reactive oxygen species in cells leading to an oxidative stress. High concentrations of ROS can lead to damage or inhibiting normal function of cellular structures, as a consequence, leading to cell death.

To counteract the adverse effects of ROS, fish have a well-developed antioxidant defense system, which includes two antioxidant categories: enzymatic (e.g., SOD, GR, GPx) and non-enzymatic (e.g., glutathione) (El-Gazzar et al. 2014). Melatonin is another non-enzymatic antioxidant (Mirończuk-Chodakowska et al. 2018). This indolamine is synthesized in the pineal gland and in other organs, such as the retina, lens, pituitary gland, bone marrow cells, gut, and skin (Acuña-Castroviejo et al. 2014). Melatonin and its metabolites are among the most effective free radical “sweepers.” What is particularly unusual is the high efficacy of melatonin as a protector against reactive oxygen (ROS) and reactive nitrogen species (RNS) (Tan et al. 2016).

These substances protect against free radicals and oxidative stress by a range of mechanisms, including direct sweeping of free radicals and their products, inducing antioxidant enzyme expression, decreasing oxidant enzyme activation, and maintaining mitochondrial homeostasis (Zhang and Zhang 2014). Moreover, melatonin has been demonstrated to decrease oxidative damage to lipids, proteins, and DNA in vivo and in vitro (Tan et al. 2016). Melatonin penetrates into cells easily (it is soluble both in aqueous solutions and in lipids), and its intracellular concentration is often higher than its concentration in body fluids. Thus, despite having a significantly lower concentration in the body than glutathione, for instance, melatonin has high availability in the cells (Karasek 2004).

Fish tissues, particularly the liver, have antioxidant defense systems to protect them from oxidative stress caused by metals (Saglam et al. 2014). There is now a vast range of studies in the literature documenting that melatonin increases the activity of antioxidant enzymes in different tissues, for example the kidneys, liver, and brain (Zhang and Zhang 2014).

To the best of our knowledge, there are no reports on the role of melatonin on the oxidative stress induced by cadmium in fish hepatopancreas (liver). Therefore, the present study aimed to (1) characterize the chronic sublethal effects of Cd exposure, i.e., the levels of cadmium, oxidative stress markers, and trace elements in the hepatopancreas and (2) evaluate the possible protective effect of melatonin against Cd toxicity in Prussian carp females.

## Materials and methods

### Experimental fish

Three hundred and forty-three Prussian carp (*Carassius gibelio* B.) females at the age of 3 years (mean body weight 204.65 ± 12.58 g and body length 23.25 ± 0.49 cm) originating from the Mydlniki Station were used in the present work. The fish were stocked in seven 700-L tanks (49 fish per tank), with constantly aerated and filtered water, under 14:10 light-dark cycle, temperature of 18 °C, dissolved oxygen concentration 9.0 mg/L, pH = 7.6 to 7.7, water hardness 186 mg CaCO_3_/L. The heavy metal concentrations in water were 0.003 mgCu /L, 0.01 mgZn/L, 0.025 mgFe/L, and 0.0045 mgCd/L. The fish were fed with commercial dry pellets during the 3-month acclimation period and throughout the experiment. These pellets were prepared from grains, oilseed, fish meal, mineral supplements, and vitamin. The feed was comprised of 37% crude protein, 12% crude fat and 32.5% carbohydrate. The fish were fed daily (3% of their body weight). After acclimation, fish were randomly assigned to seven groups: control group—nominally zero cadmium in water, group Mel—the fish received intramuscular implants containing 18 mg of melatonin (Ceva Santa Animale France), group blank—the fish were sham-injected, group 0.4 mgCd/L + Mel—the fish received intramuscular melatonin implants and were exposed to cadmium in water, group 0.4 mgCd/L—the fish were exposed to cadmium in water, group 4.0 mgCd/L + Mel—the fish received intramuscular melatonin implants and were exposed to cadmium in water, and group 4.0 mgCd/L—the fish were exposed to cadmium in water. The stock solutions of Cd were made by dissolving analytical grade of cadmium chloride (CdCl_2_·2.5H_2_O) in distilled water. The water in the tanks was exchanged every 2 days. Analysis of cadmium concentrations in water samples collected during experiment showed the following mean levels: control, Mel and blank groups—0.006 mg/L (± 0.001), group 0.4 mgCd/L + Mel—0.34 mg/L (± 0.05), group 0.4 mgCd/L—0.41 mg/L (± 0.04), group 4.0 mgCd/L + Mel—3.97 mg/L (± 0.49), group 4.0 mgCd/L 4.02 mg/L (± 0.39).

The water cadmium levels (0.4 and 4.0 mgCd/L) were selected based on the concentrations of this metal in environmental water samples, which range from 1 μg/L to over 16.1 mg/L (Tilton et al. 2003; Peng et al. 2009). A description of the procedure used to melatonin implant the fish is described by Porter et al. (1998) and Mazurais et al. (2000).

The fish were exposed to cadmium for a period of 7 or 13 weeks. After 7 weeks of exposure, each group treated with Cd was divided into two groups of fish. The groups (control, Mel, blank—not treated with Cd) were maintained under the same conditions. In the case of experimental groups, one of them was kept under the same conditions, while the second one (groups 0.4 mgCd/L + Mel-dep, 0.4 mgCd/L-dep, 4.0 mgCd/L + Mel-dep and 4.0 mgCd/L-dep) was moved to clean water for a depuration period which lasted until the end of the experiment (next 6 weeks) (Table 1).

**Table 1:** The configuration of treatment groups and cadmium doses in water during the 13-week exposure period and the following 6-week exposure or depuration period

Group	Control	Mel	blank	0.4 mgCd/L + Mel		0.4 mgCd/L		4.0 mgCd/L + Mel		0.4 mgCd/L	
Cd dose in water (mg/L) during 1–7 weeks period	–	–	–	0.4		0.4		4.0		4.0	
Number of fish at the beginning [n] (per tank)	49	49	49	49		49		49		49	
Group during 10–13 weeks of experiment	Control	Mel	Blank	0.4 mgCd/L + Mel	0.4 mgCd/L + Mel-dep	0.4 mgCd/L	0.4 mgCd/L-dep	4.0 mgCd/L + Mel	4.0 mgCd/L + Mel-dep	4.0 mgCd/L	4.0 mgCd/L-dep
Cd dose in water (mg/L) during the 10–13 weeks period	–	–	–	0.4	–	0.4	–	4.0	–	4.0	–
Number of fish at the beginning of the 10-week exposure	14/14	14/14	14/14	14	14	14	14	14	14	14	14

### Cd, Zn, Cu, and Fe determination

Samples of hepatopancreas tissues were collected in order to determine cadmium, zinc, copper, and iron concentrations after 1, 4, 7, 10, and 13 weeks of the experiment. The preparation of fish tissues for heavy metal analysis and metal determination have been represented elsewhere (Drąg-Kozak et al. 2018). Metal concentrations in the hepatopancreas were measured by atomic absorption spectrometry (Unicam 929) (Agemian et al. 1980). The results were represented in milligrams of Cd, Zn, Cu, and Fe per kilogram of wet tissue weight (ww).

### Hepatosomatic index analysis

Before the exposure and after the 1st, 4th, 7th, 10th, and 13th weeks of the experiment, the value of hepatosomatic index (HSI) was calculated according to the following formula: hepatopancreas weight (g)/whole body weight (g) × 100 (Dekić et al. 2016). To observe the relationships between HSI and Cd concentrations in liver tissue, Spearman’s correlation coefficients were calculated.

### Biochemical analysis

#### Preparation of hepatopancreas homogenates

Samples of hepatopancreas tissues before the exposure and after 1, 4, 7, 10, and 13 weeks of the experiment were collected in order to analysis of antioxidants.

Hepatopancreas samples were homogenized for 5 min in chilled phosphate buffer (0.05 M, pH 7.0) using Ultra-Turrax Model T25 homogenizer. The homogenate was centrifuged at 15,000×*g* for 15 min at 4 °C with a refrigerated centrifuge. Supernatants were used to determine the total antioxidant capacity and activities of antioxidant enzymes by using a spectrophotometric assay.

#### Antioxidants

The FRAP (ferric reducing ability of plasma) assay was used to measure the total antioxidant effect in hepatopancreas homogenates. This method is based on the reduction of ferric tripyridyltriazine (Fe^3+^–TPTZ) complex to the ferrous tripyridyltriazine (Fe^2+^) form at low pH by low-molecular-weight plasma antioxidants. Reduced Fe^2+^–TPTZ forms have an intensive blue color with an absorption maximum at 593 nm by a U-2800A UV/Vis spectrophotometer. The calibration curve was prepared with the use of five Fe^2+^ standard solutions (0.2–1.6 mmol/L) (Benzie and Strain 1996).

The level of reduced glutathione (GSH) was determined on the basis of GSH oxidation with 5.5-dithio-bis-6.2-nitrobenzoic acid using the method described by Beutler et al. (1963). This method was based upon development of stable yellow color when 5,5′-dithiobis-(2nitrobeznoic acid)-DTNB is added to sulfhydryl compounds with an absorption maximum at 415 nm. Absorbance reading was taken after a 40-s incubation period using a biochemical analyzer MaxMat PL. GSH curve was plotted by assaying different GSH standards (10–100 μmol/L) to determine the GSH concentration of the sample from the calibrator curve. The GSH concentration unit is then calculated as a micromole per gram of protein in the sample.

Glutathione reductase (GR) activity was measured by following the reduction of oxidized glutathione (GSSG) in the presence of NADPH, which is oxidized to NADP^+^. The decrease in absorbance at 340 mm was followed on the Biotek ELX808 microplate reader, and final concentrations were expressed as U/L unit.

Glutathione peroxidase (GPx) activity was measured using the method described by Paglia and Valentine (1967). In the presence of glutathione reductase and NADPH, the oxidized glutathione (GSSH) is immediately converted to reduced form with a concomitant oxidation of NADPH to NADP^+^. The decrease in NADPH absorbance measured at 340 nm on the Biotek ELX808 microplate reader and final concentrations were expressed as U/L unit.

Superoxide dismutase (SOD) activity was determined by the method described by Misra and Fridovich (1972), which is based on inhibiting of autooxidation of adrenaline to adrenochrome at alkaline pH. The reaction followed at 480 nm on the biochemical analyzer, MaxMat PL. One unit of SOD activity was defined as the amount of the enzyme that caused a 50% reduction in the autooxidation of adrenaline. The final results were expressed as units per gram of protein.

### Statistical analysis

The results of the analysis were expressed as the mean ± standard error of the mean (SEM). The results were analyzed by the means of one-way ANOVA, and the Mann-Whitney procedure was used to determine significant differences between the means for the control and experimental groups, as well as between groups at successive months of exposure. The relationship between the cadmium, zinc, copper, and iron concentrations in the liver and the dose of exposure were calculated using Spearman’s correlation coefficients. The differences between the means were determined as significant for *p* ≤ 0.05.

## Results

### Cd accumulation in hepatopancreas

The results of the cadmium level analysis in the hepatopancreas of Prussian carp, which were collected after the 1st, 4th, 7th, 10th, and 13th weeks of the exposure to different concentrations of cadmium, are presented in Table 3. A statistically significant (*p* < 0.05) increase in Cd levels in the hepatopancreas compared to sample 0 (baseline) was found as soon as the first week of exposure, and this persisted until the end of the exposure period. A statistically significant positive correlation coefficient confirmed that the accumulation was correlated with the Cd dose (Table 3). The maximum cadmium concentration occurred after the tenth week of the exposure (Table 3). The maximum Cd concentration was found after the tenth week of exposure. In the group of females exposed to the highest Cd doses (4 mgCd/L), Cd levels were significantly higher (*p* < 0.05) than in the controls and other groups, at all measurements. In the group of fish with melatonin implants (4.0 mgCd/L + Mel), Cd levels in the hepatopancreas were significantly lower (*p* < 0.05) at all measurements. Spearman’s correlation coefficients were calculated to determine the relationship between Cd concentration in water and in hepatopancreas during exposure. Positive correlations were noted from the fourth week to the end of the exposure (Table 3).

Cd concentration analysis results for the 7 weeks of exposure and 6 weeks of depuration are shown in Table 3. After 3 weeks of depuration, there was a statistically significant decrease (*p* < 0.05) in Cd levels in the hepatopancreas of fish previously exposed to the highest doses of the metal. During the depuration phase, Spearman’s correlation coefficients were significantly positive confirming the relationship between the Cd hepatopancreas level and its previous exposure level (Table 3).

In the 4.0 mgCd/L + Mel-dep group, a statistically significant decrease in Cd in the hepatopancreas was found both after 3 and 6 weeks of depuration (Table 3). A positive correlation between Cd hepatopancreas concentration to cadmium doses was observed after third and sixth week of the depuration period (Table 3).

### Zn accumulation in hepatopancreas

The results of the zinc concentration analysis in the hepatopancreas of Prussian carp are shown in Table 2. In the group of fish exposed to the highest dose of Cd, there was a statistically significant increase (*p* < 0.05) in Zn levels in the hepatopancreas at 4, 7, and 10 weeks of exposure compared to the remaining groups. A high, statistically significant correlation coefficient confirms that Zn levels depended on the Cd dose in the exposure period (Table 2). In the 4.0 mgCd/L + Mel group, there was a statistically significant decrease (*p* < 0.05) in Zn levels in the hepatopancreas at 4, 7, and 10 weeks of exposure compared to the 4.0 mgCd/L group. A positive correlation between Cd and Zn concentrations in the hepatopancreas was observed from the fourth week until the end of the exposure (Table 2).

**Table 2:** Comparison of Zn, Cu, and Fe concentration (mg/kg) in the hepatopancreas of female Prussian carp during 13 weeks of fish exposure to different doses of cadmium and 6 weeks depuration. Spearman’s correlation coefficients (*r*) for the relationship of Zn, Cu, and Fe hepatopancreas concentration to cadmium doses during the exposure and depuration periods in different groups. The results of multiple comparisons Kruskal-Wallis test (*p*) for Zn concentration in hepatopancreas, value difference

				Exposure			Zn in hepatopancreas (mg/kg)					Depuration			
Week	Control	Mel	Blank	0.4 mgCd/L + Mel	0.4 mgCd/L	4.0 mgCd/L + Mel	4.0 mgCd/L	*r*	Statistics	0.4 mgCd/L + Mel-dep	0.4 mgCd/L-dep	4.0 mgCd/L + Mel-dep	4.0 mgCd/L-dep	*r*	Statistics
0	16.77 ± 0.84Aa	16.63 ± 0.75Aa	17.34 ± 0.77Aa	17.06 ± 0.75Aa	16.92 ± 0.82Aa	17.20 ± 0.75Aa	17.34 ± 0.72Aa	ns	ns	NT	NT	NT	NT	NT	NT
1	18.93 ± 1.59ADab	19.53 ± 2.16BCDabcd	16.51 ± 1.60ABa	24.91 ± 1.43Cb	12.80 ± 0.58Bb	22.87 ± 1.59Dbd	21.06 ± 0.97Db	0.32*	*p* < 0.0009	NT	NT	NT	NT	NT	NT
4	20.67 ± 1.16Ab	17.11 ± 0.88Bad	17.28 ± 3.23ABac	19.71 ± 0.90ABc	20.96 ± 1.13Ac	19.95 ± 0.99ABab	31.13 ± 2.52Cc	0.53***	*p* < 0.0016	NT	NT	NT	NT	NT	NT
7	25.60 ± 1.15ACc	23.58 ± 1.13Ab	29.28 ± 1.70ABCb	31.71 ± 1.88Bd	31.37 ± 2.31BCd	43.81 ± 0.97Dc	53.13 ± 3.13Ed	0.83***	*p* < 0.0001	NT	NT	NT	NT	NT	NT
10	18.09 ± 0.90ACab	14.34 ± 1.06Bc	28.00 ± 1.48Ccb	21.30 ± 1.12Dbc	24.31 ± 1.45CDcd	35.96 ± 2.42Ee	45.32 ± 4.04Fde	0.58***	*p* < 0.0001	21.21 ± 0.98Db	28.27 ± 2.53Ca	28.64 ± 1.44Cb	100.4 ± 6.29Db	0.54***	*p* < 0.0001
13	16.94 ± 1.12Aab	19.56 ± 0.81Ad	29.94 ± 1.95Bb	20.65 ± 1.14Ab	26.34 ± 1.28Bd	38.41 ± 1.81Cde	37.25 ± 2.23CDe	0.57***	*p* < 0.0001	15.87 ± 1.01Cc	15.68 ± 1.18Cb	28.53 ± 1.94Bb	37.70 ± 1.66Dc	0.33*	*p* < 0.0001
				Exposure			Cu in hepatopancreas (mg/kg)					Depuration			
0	7.62 ± 0.55Aa	7.61 ± 0.55Aa	7.61 ± 0.55Aa	7.62 ± 0.55Aac	7.61 ± 0.55Aa	7.62 ± 0.55Aa	7.62 ± 0.55Aa	ns	ns	NT	NT	NT	NT	NT	NT
1	6.74 ± 0.36ACa	9.40 ± 0.68BDa	5.69 ± 0.31Ab	9.05 ± 0.26Bab	6.75 ± 0.19Ca	11.41 ± 0.68Dc	8.38 ± 0.52Bab	0.59***	*p* < 0.0001	NT	NT	NT	NT	NT	NT
4	5.85 ± 0.40ACab	4.73 ± 0.40Ab	7.25 ± 0.54CBab	8.38 ± 0.44Ba	8.24 ± 0.61BDa	7.32 ± 0.44BCa	9.72 ± 0.31Dbc	0.50***	*p* < 0.0001	NT	NT	NT	NT	NT	NT
7	6.70 ± 0.21Aa	7.62 ± 0.39Ba	10.02 ± 0.42Cc	9.80 ± 0.59Ca	11.49 ± 1.02Cb	10.09 ± 0.53Cbc	10.86 ± 0.63Cc	0.58***	*p* < 0.0001	NT	NT	NT	NT	NT	NT
10	6.43 ± 0.35ADab	5.05 ± 0.25Bb	6.38 ± 0.37Aab	6.53 ± 0.22ABc	7.88 ± 0.43Ca	7.82 ± 0.53Dab	12.27 ± 1.11Ed	0.66***	*p* < 0.0001	6.55 ± 0.45Ab	7.16 ± 0.38Ab	8.25 ± 0.66Aab	16.16 ± 0.25Cb	0.60***	*p* < 0.0001
13	5.69 ± 0.18Ab	5.98 ± 0.46ABb	7.03 ± 0.31Ca	4.53 ± 0.48Ab	6.50 ± 0.18BCc	4.49 ± 0.56Aa	6.02 ± 0.35Ae	− 0.2919*	*p* < 0.0006	4.83 ± 0.30DCc	4.23 ± 0.16Cc	6.37 ± 0.27ABb	5.66 ± 0.25Ac	ns	*p* < 0.0001
				Exposure			Fe in hepatopancreas (mg/kg)					Depuration			
0	77.78 ± 4.99Aad	73.50 ± 3.12Aad	73.50 ± 4.61Aa	74.93 ± 4.11Aa	80.64 ± 3.97Aa	76.36 ± 4.43Aac	72.07 ± 3.59Aac	ns	ns	NT	NT	NT	NT	NT	NT
1	107.5 ± 9.95ACb	116.9 ± 7.05Ab	64.84 ± 4.80Bac	87.29 ± 5.87Cac	53.82 ± 2.90Bb	111.2 ± 6.78Ab	84.31 ± 3.92Da	ns	*p* < 0.0001	NT	NT	NT	NT	NT	NT
4	79.90 ± 4.79ACa	73.27 ± 4.66Aa	73.31 ± 5.25ACa	100.9 ± 6.66BCc	99.62 ± 6.04Bc	89.70 ± 4.81Ca	76.03 ± 5.53ACac	0.31*	*p* < 0.0058	NT	NT	NT	NT	NT	NT
7	68.23 ± 3.08Adc	79.73 ± 3.88Bd	102.0 ± 4.31CDb	97.08 ± 6.05BCc	94.42 ± 7.43BCac	120.3 ± 7.02Db	108.2 ± 5.30CDb	0.56***	*p* < 0.0001	NT	NT	NT	NT	NT	NT
10	57.66 ± 4.81Ac	38.33 ± 2.75Bc	79.99 ± 4.09Ca	44.27 ± 2.28Bb	62.51 ± 3.28Ad	63.28 ± 3.61Ac	116.7 ± 7.14Db	0.29*	*p* < 0.0001	56.97 ± 2.55Db	67.01 ± 4.62CDb	65.76 ± 3.21Db	124.4 ± 6.38Ea	0.39**	*p* < 0.0001
13	37.48 ± 2.41Ae	41.12 ± 3.80ACc	51.98 ± 3.20BDc	44.82 ± 4.19ABb	49.09 ± 0.80Db	62.51 ± 6.97BCc	61.92 ± 3.84Bc	0.45**	*p* < 0.0005	30.21 ± 1.88Dc	31.62 ± 2.59ADc	45.92 ± 1.65BCc	67.26 ± 6.85Eb	ns	*p* < 0.0001

Capital letters denote statistically significant differences (*p* < 0.05) between the groups in different exposure weeks, while small letters indicate significant differences in the groups between successive weeks of the exposure

*NT* not tested, *ns* not significant

*p < 0.05; **p < 0.01; ***p < 0.001

Zn concentration analysis results for the 7 weeks of exposure and 6 weeks of subsequent depuration are shown in Table 2. After 3 weeks of depuration, there was a statistically significant increase (*p* < 0.05) in Zn concentration in the hepatopancreas of fish previously exposed to the highest doses of the metal, up to 100.0 ± 6.29 mgZn/kg. A statistically significant positive correlation coefficient confirms that Zn levels depended on the Cd dose in the exposure period (Table 2). In the 4.0 mgCd/L + Mel-dep group, a statistically significant decrease in Zn levels was found in the hepatopancreas of fish previously exposed to the highest doses of Cd, both after 3 and 6 weeks of depuration (Table 2). A positive correlation between Cd and Zn concentration in the hepatopancreas was noted after 3- and 6-week depuration periods (Table 2).

### Cu accumulation in hepatopancreas

The results of copper concentration analysis in the hepatopancreas of Prussian carp are shown in Table 2. After 1, 4, 7, and 10 weeks of exposure, a statistically significant increase (*p* < 0.05) in Cu levels was found in the hepatopancreas of fish exposed to the highest doses of Cd (4.0 mgCd/L) compared to the controls. Statistically significant coefficients of correlation confirmed the association with the Cd dose (Table 2). After 4 and 10 weeks of exposure, there was a statistically significant increase (*p* < 0.05) in Cu levels in groups exposed to Cd (0.4 and 4.0 mgCd/L) compared to groups of fish treated with melatonin (0.4 mgCd/L + Mel and 4.0 mgCd/L + Mel), with the latter found to have a significantly lower Cu concentration in the hepatopancreas. Cu concentration analysis results for the 7 weeks of exposure and 6 weeks of depuration are shown in Table 2. After 3 weeks of depuration, there was a statistically significant (*p* < 0.05) increase in Cu concentration in the hepatopancreas of fish previously exposed to the highest doses of Cd compared to other groups. In the 4.0 mgCd/L + Mel-dep group, there was a statistically significant decrease in Cu levels in the hepatopancreas (Table 2). A positive correlation between Cd and Cu concentration in the hepatopancreas was noted in the 3-week depuration period (Table 2).

### Fe accumulation in hepatopancreas

The results of the iron concentration analysis in the hepatopancreas of Prussian carp are reported in Table 2. After 1 week of exposure to Cd (0.4 and 4.0 mgCd/L), there was a statistically significant decrease (*p* < 0.05) in Fe concentration compared to groups treated with melatonin (0.4 mgCd/L + Mel and 4.0 mgCd/L + Mel) or the controls. In the 0.4 mgCd/L + Mel and 4.0 mgCd/L + Mel groups, the Fe levels in the hepatopancreas were significantly higher (*p* < 0.05) (Table 2). After 10 weeks of exposure, there was a statistically significant increase (*p* < 0.05) in Fe levels in groups exposed to Cd (0.4 and 4.0 mgCd/L) compared to groups treated with melatonin (0.4 mgCd/L + Mel and 4.0 mgCd/L + Mel) (Table 2). Spearman’s positive correlation was observed after a 4-week period to the end of exposure (Table 2).

After 3 weeks of depuration, there was a statistically significant increase (*p* < 0.05) in Fe concentration in the hepatopancreas of fish previously exposed to the highest doses of Cd compared to other groups. A statistically significant positive correlation coefficient confirms that Fe levels were associated with the Cd dose in the exposure period (Table 2). In the 4.0 mgCd/L + Mel-dep group, a statistically significant decrease in Fe levels was found in the hepatopancreas of fish previously exposed to the highest doses of Cd, both after 3 and 6 weeks of depuration (Table 2).

### Hepatosomatic index analysis

Fish in the 0.4 mgCd/L + Mel, 4.0 mgCd/L + Mel, and 4.0 mgCd/L groups had a significantly lower HSI (*p* < 0.05) after 7 weeks of exposure. After 10 and 13 weeks of exposure to the highest dose of Cd, HSI was lower than in other groups (Table 3). A negative correlation between HSI and Cd concentration in the hepatopancreas was observed after a 4-week period to the end of exposure.

**Table 3:** Comparison of Cd concentration (mg/kg) and the hepatosomatic index (HSI) of female Prussian carp during 13 weeks of fish exposure to different doses of cadmium and 6 weeks depuration. Spearman’s correlation coefficients (*r*) for the relationship of Cd hepatopancreas concentration to cadmium doses during the exposure and depuration periods in different groups. Comparison of Spearman’s correlation coefficients (*r*) for the relationship between HSI value and Cd concentration in hepatopancreas. The results of multiple comparisons Kruskal-Wallis test (*p*) for HSI values differences

			Exposure				Cd in hepatopancreas (mg/kg)					Depuration			
Week	Control	Mel	Blank	0.4 mgCd/L + Mel	0.4 mgCd/L	4.0 mgCd/L + Mel	4.0 mgCd/L	*r*	Statistics	0.4 mgCd/L + Mel-dep	0.4 mgCd/L-dep	4.0 mgCd/L + Mel-dep	4.0 mgCd/L-dep	*r*	Statistics
0	0.04 ± 0.001Aac	0.04 ± 0.002Aa	0.03 ± 0.001Aa	0.04 ± 0.002Aa	0.04 ± 0.002Aa	0.03 ± 0.00Aa	0.04 ± 0.001Aa	ns	ns	NT	NT	NT	NT	NT	NT
1	0.03 ± 0.001Aa	0.04 ± 0.001Bb	0.01 ± 0.001Cb	0.17 ± 0.01Db	0.13 ± 0.01Db	1.47 ± 0.10Eb	1.83 ± 0.17Fb	0.94***	*p* < 0.0001	NT	NT	NT	NT	NT	NT
4	0.03 ± 0.01ACac	0.05 ± 0.01Ab	0.02 ± 0.001Cab	2.05 ± 0.21Bc	2.44 ± 0.27Bc	9.00 ± 0.44Dc	10.73 ± 0.37Ec	0.94***	*p* < 0.0001	NT	NT	NT	NT	NT	NT
7	0.08 ± 0.01Ab	0.05 ± 0.01Bb	0.08 ± 0.01Ac	1.34 ± 0.10Cd	3.36 ± 0.22Dc	15.64 ± 0.4Ed	29.87 ± 1.25Fd	0.94***	*p* < 0.0001	NT	NT	NT	NT	NT	NT
10	0.10 ± 0.05Abc	0.10 ± 0.01Ac	0.06 ± 0.01Bd	3.21 ± 0.28Ce	2.65 ± 0.29Cc	26.63 ± 2.84De	45.43 ± 2.74Ee	0.94***	*p* < 0.0001	2.36 ± 0.32Cb	5.97 ± 0.47Db	4.05 ± 0.74Db	10.15 ± 0.25Cb	0.86***	*p* < 0.0001
13	0.05 ± 0.01Ac	0.08 ± 0.01Bc	0.04 ± 0.01Ae	4.67 ± 0.42Cf	3.01 ± 0.30Dc	22.95 ± 0.99Ee	25.13 ± 1.38Ed	0.94***	*p* < 0.0001	0.99 ± 0.08Dc	0.61 ± 0.08Cc	2.67 ± 0.32Eb	14.49 ± 0.91Ec	0.94***	*p* < 0.0001
				Exposure			HSI (%)					Depuration			
0	4.86 ± 0.56Aa	4.88 ± 0.57Aa	4.91 ± 0.55Aa	4.61 ± 0.48Aac	4.56 ± 0.57Aa	4.41 ± 0.46Aac	5.03 ± 0.54Aac	ns	ns	NT	NT	NT	NT	NT	NT
1	3.92 ± 0.52Aa	4.04 ± 0.57ABa	5.62 ± 0.70ABa	5.33 ± 0.41ABa	5.98 ± 0.59Bab	4.72 ± 0.22ABa	4.03 ± 0.31Aab	ns	*p* < 0.021	NT	NT	NT	NT	NT	NT
4	4.06 ± 0.34Aa	4.48 ± 0.39Aa	5.12 ± 0.63Aa	3.97 ± 0.44Aa	4.59 ± 0.42Aa	3.84 ± 0.32Ac	3.99 ± 0.36Aab	− 0.3054*	ns	NT	NT	NT	NT	NT	NT
7	4.09 ± 0.21Aa	5.11 ± 0.20Ba	3.27 ± 0.24Cb	3.98 ± 0.23ACc	4.60 ± 0.55ABa	3.51 ± 0.32ACc	3.62 ± 0.28ACb	− 0.34*	*p* < 0.019	NT	NT	NT	NT	NT	NT
10	6.91 ± 0.40Ab	7.23 ± 0.57ABb	6.44 ± 0.70ABCa	8.22 ± 0.21Bb	7.06 ± 0.52Abc	5.79 ± 0.43ACa	5.36 ± 0.43Cc	− 0.30*	*p* < 0.012	6.44 ± 0.47Ab	7.46 ± 0.64Ab	5.93 ± 0.48Ab	3.77 ± 0.39Ba	− 0.32*	*p* < 0.0034
13	9.53 ± 0.62ACc	10.05 ± 0.48Ac	3.27 ± 0.24ACc	9.23 ± 0.81ACb	8.63 ± 0.30Cc	6.93 ± 0.24Bb	6.43 ± 0.65Bc	− 0.53***	*p* < 0.0001	9.80 ± 0.64Ac	10.43 ± 0.76Ac	8.82 ± 0.45Ac	6.05 ± 0.79Bb	− 0.31*	*p* < 0.0001

Capital letters denote statistically significant differences (*p* < 0.05) between the groups in different exposure weeks, while small letters indicate significant differences in the groups between successive weeks of the exposure

*NT* not tested, *ns* not significant

*p < 0.05; **p < 0.01; ***p < 0.001

After the 10th and 13th weeks of sampling, a statistically significant (*p* < 0.05) increase in HSI values was noted in all groups compared to the previous periods (Table 3). This might have been caused by spawning, which took place in the seventh week of exposure (Table 3).

After 1 and 2 weeks of depuration, HSI was significantly lower (*p* < 0.05) in the group exposed to the highest dose of Cd than in the remaining groups (Table 3). Among fish with melatonin implants, during depuration, HSI was significantly higher in fish previously exposed to the highest dose of Cd (Table 3).

A negative correlation between HSI and Cd concentration in the hepatopancreas was observed after a 3- and 6-week period (Table 3).

### Antioxidants

#### Total antioxidant status

Total antioxidant status of the liver tissue was measured in terms of FRAP. After 4 weeks of exposure, FRAP levels in the hepatopancreas were significantly lower (*p* < 0.05) in groups exposed to Cd (0.4 and 4.0 mgCd/L) compared to groups of fish treated with melatonin (0.4 mgCd/L + Mel and 4.0 mgCd/L + Mel) and the controls. After 10 and 13 weeks of exposure, FRAP levels were also lower in the group exposed to the highest dose of Cd compared to the group treated with melatonin, as confirmed by statistically significant correlation coefficients (Table 4).

**Table 4:** Effect of cadmium, melatonin, and their combination on level of FRAP (μmol/L) and GSH (μmol/L) in the hepatopancreas of female Prussian carp during 13 weeks of fish exposure to different doses of cadmium and 6 weeks depuration. Spearman’s correlation coefficients (*r*) for the relationship of FRAP hepatopancreas concentration to cadmium doses during the exposure and depuration periods in different groups. The results of multiple comparisons Kruskal-Wallis test (*p*) for FRAP and GSH level in hepatopancreas, value difference

				Exposure			FRAP (μmol/L)					Depuration			
Week	Control	Mel	Blank	0.4 mgCd/L + Mel	0.4 mgCd/L	4.0 mgCd/L + Mel	4.0 mgCd/L	*r*	Statistics	0.4 mgCd/L + Mel-dep	0.4 mgCd/L-dep	4.0 mgCd/L + Mel-dep	4.0 mgCd/L-dep	*r*	Statistics
0	834.7 ± 53.5Aa	872.6 ± 54.1Aa	799.2 ± 56.1Aa	837.2 ± 41.2Aac	832.1 ± 41.6Aa	837.2 ± 41.2Aa	837.2 ± 41.2Aa	ns	ns	NT	NT	NT	NT	NT	NT
1	911.5 ± 47.3ACDa	995.2 ± 61.4Aa	896.0 ± 49.2ABCDa	721.4 ± 46.8ABab	846.8 ± 29.5Aa	829.2 ± 36.3Ca	1036.0 ± 38.0Db	ns	*p* < 0.0026	NT	NT	NT	NT	NT	NT
4	920.7 ± 66.0Aa	264.9 ± 34.5BCDac	80.53 ± 4.55Bb	764.3 ± 47.4Aa	133.1 ± 8.97Cb	819.9 ± 36.9Aa	366.4 ± 39.9Dce	ns	*p* < 0.0001	NT	NT	NT	NT	NT	NT
7	837.7 ± 45.5Aa	849.0 ± 44.0Aa	1156.0 ± 66.3Bc	970.6 ± 42.5ABc	1044.0 ± 60.1Bc	893.2 ± 71.2Aa	925.6 ± 46.9Aab	ns	*p* < 0.0109	NT	NT	NT	NT	NT	NT
10	446.7 ± 38.0ADb	303.3 ± 28.9Bb	388.3 ± 30.9ABd	604.2 ± 30.9Cb	465.7 ± 23.1ADd	661.9 ± 43.0Cb	498.3 ± 24.3Dd	0.57***	*p* < 0.0001	524.7 ± 531ACb	595.8 ± 350Cb	565.6 ± 18.2Cb	931.9 ± 49.8Da	0.76***	*p* < 0.0001
13	428.1 ± 28.6ACb	421.0 ± 18.5Ac	466.0 ± 20.3ACd	477.0 ± 22.5ACd	520.2 ± 21.1Cd	566.3 ± 19.4Bb	440.0 ± 24.8Aed	0.04*	*p* < 0.0034	518.8 ± 15.1Bb	526.5 ± 25.7CBb	448.5 ± 42.5ABb	616.5 ± 24.3Db	0.47***	*p* < 0.0006
				Exposure			GSH (μmol/L)					Depuration			
0	191.3 ± 12.23Aac	178.5 ± 14.5Aa	177.0 ± 9.45Aa	198.7 ± 9.30Aac	191.3 ± 16.73Aa	188.5 ± 11.81Aa	178.5 ± 6.09Aa	ns	ns	NT	NT	NT	NT	NT	NT
1	183.7 ± 10.68Aa	230.9 ± 9.47Bb	209.1 ± 13.2ABac	178.2 ± 14.4Aa	217.8 ± 19.75Aab	196.1 ± 14.69ABa	211.8 ± 10.01ABb	ns	ns	NT	NT	NT	NT	NT	NT
4	202.3 ± 9.26ACDac	181.8 ± 8.60ADa	242.9 ± 8.58Bb	233.4 ± 12.97BCc	235.9 ± 8.84Bb	183.7 ± 5.17Da	268.3 ± 20.56Bc	ns	*p* < 0.0002	NT	NT	NT	NT	NT	NT
7	243.5 ± 9.83ADb	179.1 ± 8.27Ba	225.2 ± 10.64ACDbc	198.1 ± 10.77Bac	247.0 ± 15.57ADbc	145.2 ± 11.34Cb	249.6 ± 11.36Dbc	ns	*p* < 0.0001	NT	NT	NT	NT	NT	NT
10	218.9 ± 7.92Abc	172.4 ± 10.14BDa	201.9 ± 5.01Bc	282.7 ± 12.78Cb	239.3 ± 10.83Abc	156.3 ± 5.52Db	251.3 ± 13.84Ac	ns	*p* < 0.0001	218.1 ± 10.31a	247.7 ± 12.99ACa	277.0 ± 13.67Cb	269.7 ± 17.37Ca	0.64***	*p* < 0.0364
13	357.8 ± 12.54Ad	259.7 ± 15.67BCb	280.4 ± 5.77Bd	222.3 ± 14.91Cbc	282.4 ± 11.91Bc	303.1 ± 15.36Bc	369.1 ± 15.95Ad	ns	*p* < 0.0001	363.0 ± 12.16Ab	298.4 ± 15.63Bb	265.2 ± 11.99Bb	269.1 ± 14.16Ba	ns	*p* < 0.0001

Capital letters denote statistically significant differences (*p* < 0.05) between the groups in different exposure weeks, while small letters indicate significant differences in the groups between successive weeks of exposure

*NT* not tested, *ns* not significant

*p < 0.05; **p < 0.01; ***p < 0.001

After 3 and 6 weeks of depuration, FRAP was significantly higher (*p* < 0.05) in the group previously exposed to the highest dose of Cd than in the remaining groups. In the 4.0 mgCd/L + Mel-dep group, there was a statistically significant decrease (*p* < 0.05) of FRAP levels in the hepatopancreas compared to the group previously exposed to the highest dose of Cd (Table 4). A positive correlation between FRAP and Cd concentration in the hepatopancreas was observed after a 3- and 6-week period (Table 4).

#### Reduced glutathione

Glutathione (GSH) levels in the hepatopancreas of fish exposed to the highest dose of Cd were significantly higher (*p* < 0.05) after 4 weeks of exposure compared to the controls. After 4, 7, 10, and 13 weeks of exposure, the 4.0 mgCd/L + Mel group had significantly lower (*p* < 0.05) GSH levels compared to the 4.0 mgCd/L group. The Spearman’s correlation coefficients were not significant (Table 4).

After 3 weeks of depuration, there was a statistically significant difference between the group exposed to the highest Cd dose and the controls, with statistically significant correlation coefficients (Table 4).

#### Glutathione reductase

After 10 weeks of exposure, glutathione reductase (GR) activity was significantly higher (*p* < 0.05) in the hepatopancreas of fish previously exposed to the highest dose of Cd than in the remaining groups. After 10 weeks of exposure, the 4.0 mgCd/L + Mel group had significantly lower (*p* < 0.05) GR activity compared to the 4.0 mgCd/L group (Table 5).

**Table 5:** Effect of cadmium, melatonin and their combination on level of GR (U/L), GPx (U/mL), and SOD (U/L) in the hepatopancreas of female Prussian carp during 13 weeks of fish exposure to different doses of cadmium and 6 weeks depuration. Spearman’s correlation coefficients (*r*) for the relationship of Fe hepatopancreas concentration to cadmium doses during the exposure and depuration periods in different groups. The results of multiple comparisons Kruskal-Wallis test (*p*) for GR, GPx and SOD level in hepatopancreas, value difference

				Exposure			GR (U/L)					Depuration			
Week	control	Mel	Blank	0.4 mgCd/L + Mel	0.4 mgCd/L	4.0 mgCd/L + Mel	4.0 mgCd/L	*r*	Statistics	0.4 mgCd/L + Mel-dep	0.4 mgCd/L-dep	4.0 mgCd/L + Mel-dep	4.0 mgCd/L-dep	*r*	Statistics
0	422.3 ± 29.22Aa	408.0 ± 35.29Aac	415.1 ± 28.33Ac	415.1 ± 13.13Aa	422.3 ± 12.53Aa	410.8 ± 11.62Aa	413.7 ± 11.84Aa	ns	ns	NT	NT	NT	NT	NT	NT
1	415.9 ± 21.24ABa	493.1 ± 21.25Aa	462.7 ± 29.90Aa	428.6 ± 28.75ABa	389.1 ± 16.15Bab	426.5 ± 36.20ABa	388.8 ± 15.18Bab	ns	ns	NT	NT	NT	NT	NT	NT
4	384.3 ± 19.03ACab	323.4 ± 14.60Bc	330.0 ± 22.78ABb	391.6 ± 15.41ACa	373.8 ± 16.65Ab	424.6 ± 21.43Ca	414.6 ± 24.28ACab	0.45**	*p* < 0.0104	NT	NT	NT	NT	NT	NT
7	330.3 ± 21.10Ab	412.4 ± 15.48Bc	458.6 ± 25.51Ba	377.1 ± 25.10ABa	393.5 ± 34.42ABab	365.7 ± 17.14Aa	355.2 ± 19.10Ab	ns	*p* < 0.0211	NT	NT	NT	NT	NT	NT
10	243.2 ± 12.63Ac	177.1 ± 23.14ABb	226.8 ± 15.08Ac	167.7 ± 4.91Bb	211.7 ± 10.46Ac	226.0 ± 19.58Ab	297.6 ± 13.28Cc	ns	*p* < 0.0003	255.9 ± 27.20Ab	299.2 ± 29.85ABb	234.2 ± 17.51Ab	374.1 ± 17.60Ba	0.57***	*p* < 0.0009
13	162.8 ± 11.86ABd	156.0 ± 14.95ABb	144.2 ± 11.04Ad	217.4 ± 29.28Bc	188.2 ± 21.35ABc	203.8 ± 21.20Bb	164.8 ± 15.46ABd	0.29*	ns	171.5 ± 11.54Ac	113.3 ± 10.72Bc	162.5 ± 16.69Ac	189.5 ± 22.85Ab	ns	ns
				Exposure			GPx (U/mL)					Depuration			
0	5092 ± 241.5Aa	5006 ± 219.1Aa	4992 ± 214.7Aa	5005 ± 213.2Aa	4940 ± 190.2Aa	4997 ± 202.0Aa	4969 ± 226.5Aa	ns	ns	NT	NT	NT	NT	NT	NT
1	3662 ± 138.0Ab	6233 ± 438.6Bb	3963 ± 151.0Ab	5861 ± 189.0Bb	2317 ± 132.5Cb	5095 ± 317.6Bb	2233 ± 144.9Cb	ns	*p* < 0.0001	NT	NT	NT	NT	NT	NT
4	2869 ± 172.7Ab	3093 ± 199.2Ac	2757 ± 170.7ABc	3131 ± 367.5Ac	3161 ± 164.6Ac	1596 ± 102.2Cc	2275 ± 151.8Bb	− 0.46***	*p* < 0.0002	NT	NT	NT	NT	NT	NT
7	4019 ± 144.6Acd	4069 ± 232.7Ad	3141 ± 183.9Bc	3223 ± 177.2Bc	3224 ± 352.8ABc	3122 ± 223.1Bd	2306 ± 130.9Cb	− 0.61***	*p* < 0.0003	NT	NT	NT	NT	NT	NT
10	4132 ± 102.4Ac	4065 ± 69.25ABd	4047 ± 88.98ABd	4538 ± 180.9Ad	4120 ± 72.84Ad	3720 ± 143.0Bde	3934 ± 157.4ABd	ns	ns	3987 ± 71.61Ab	4030 ± 202.2Ab	4431 ± 53.53Bb	2780 ± 135.1Ca	ns	*p* < 0.0022
13	3620 ± 145.5Ad	4182 ± 120.8BCd	4356 ± 57.11Be	3563 ± 269.3ACc	3608 ± 259.5ACc	3834 ± 130.4ACe	3977 ± 77.53Cd	ns	*p* < 0.0029	4067 ± 76.64Cb	4066 ± 228.5BCb	4322 ± 193.5BCb	4730 ± 224.0Db	0.35*	*p* < 0.0016
SOD (U/L)				Exposure								Depuration			
0	30.80 ± 2.60Aa	31.09 ± 2.80Aa	31.09 ± 2.80Aa	31.09 ± 2.38Aa	31.23 ± 2.77Aac	30.09 ± 2.84Aa	31.09 ± 2.58Aad	ns	ns	NT	NT	NT	NT	NT	NT
1	54.29 ± 3.04Ab	56.67 ± 3.94Ab	41.99 ± 1.17Bb	51.41 ± 2.57Ab	27.74 ± 1.73Ca	56.54 ± 3.10Ab	40.2 ± 2.23Bab	ns	*p* < 0.0001	NT	NT	NT	NT	NT	NT
4	42.94 ± 2.57ABCc	42.21 ± 1.94ABCc	39.63 ± 2.81ACab	37.11 ± 2.77Aacd	50.60 ± 3.47Bb	47.91 ± 3.13Cbc	37.69 ± 2.24Aab	ns	*p* < 0.0318	NT	NT	NT	NT	NT	NT
7	39.73 ± 1.96Ac	43.40 ± 1.78Ac	44.31 ± 1.78Ab	43.51 ± 1.78Abcd	38.96 ± 2.48Ad	41.46 ± 1.64Ac	40.66 ± 1.96Ab	ns	ns	NT	NT	NT	NT	NT	NT
10	44.15 ± 2.55ADc	37.23 ± 2.26ABc	38.42 ± 1.91ABDab	34.71 ± 1.91Ba	67.51 ± 1.61Ce	44.31 ± 1.70Dc	81.80 ± 6.52Ec	0.48***	*p* < 0.0001	61.82 ± 5.88Bb	47.10 ± 1.81Cb	39.63 ± 1.84Aab	50.83 ± 3.00Cb	0.45**	*p* < 0.0001
13	40.77 ± 1.71ACc	37.69 ± 1.25Ac	44.76 ± 1.82Cb	40.54 ± 3.42ACc	35.74 ± 1.54Acd	34.49 ± 0.56Bd	29.97 ± 1.24Dd	− 0.67***	*p* < 0.0001	37.11 ± 1.68Bc	39.98 ± 1.77ABCa	35.90 ± 0.78Bb	37.91 ± 1.22ABa	−0.45**	*p* < 0.0059

Capital letters denote statistically significant differences (*p* < 0.05) between the groups in different exposure weeks, while small letters indicate significant differences in the groups between successive weeks of exposure

*NT* not tested, *ns* not significant

*p < 0.05; **p < 0.01; ***p < 0.001

After 3 weeks of depuration, GR activity was significantly increased (*p* < 0.05) in the group previously exposed to the highest dose of Cd. The 4.0 mgCd/L + Mel group had significantly lower (*p* < 0.05) GR activity compared to the 4.0 mgCd/L group (Table 5). A significant positive correlation between GR activity and Cd concentration in the hepatopancreas was observed after a 3-week depuration period (Table 5).

#### Glutathione peroxidase

After 1 week of exposure, there was a statistically significant decrease (*p* < 0.05) in glutathione peroxidase (GPx) activity in the hepatopancreas of fish exposed to either dose of Cd compared to the remaining groups. In the groups with melatonin implants, GPx activity was significantly higher than in other groups (Table 5). After 4 and 7 weeks, there was a statistically significant decrease (*p* < 0.05) in GPx activity in the group exposed to the highest Cd dose compared to the remaining groups, with statistically significant correlation coefficients (Table 5).

After 3 weeks of depuration, there was a decrease in GPx activity in the hepatopancreas of fish exposed to the highest dose of Cd compared to the remaining groups (Table 5). The 4.0 mgCd/L + Mel group had significantly higher (*p* < 0.05) GPx activity, compared to the 4.0 mgCd/L group. After 6 weeks of depuration, GPx activity increased significantly in the group previously exposed to the highest dose of Cd compared to controls. A significant positive correlation between GPx activity and Cd concentration in the hepatopancreas was observed after a 6-week depuration period (Table 5).

#### Superoxide dismutase

After the first week of exposure, in the Cd-exposed groups there was a statistically significant decrease (*p* < 0.05) of superoxide dismutase (SOD) activity compared to other groups. In the Cd + Mel groups, there was a statistically significant (*p* < 0.05) increase in SOD activity. A statistically significant decrease was found in the group exposed to the highest Cd dose, compared to other groups after 13 weeks of exposure. After 10 weeks of exposure, there was a statistically significant increase (*p* < 0.05) of SOD activity in the groups exposed to Cd (0.4 mg/L and 4.0 mg/L) compared to others. In groups exposed to Cd and treated with melatonin (0.4 mg/L + Mel and 4.0 mg/L + Mel), SOD activity was lower than in groups exposed to Cd only (both 0.4 mg/L and 4.0 mg/L). Significant positive and negative correlations between SOD activity and Cd concentration in the hepatopancreas were observed after a 10- and 13-week period, respectively (Table 5).

After 3 weeks of depuration, in the 0.4 mg/L + Mel-dep group, there was a statistically significant increase (*p* < 0.05) in SOD activity compared to other groups. In turn, in the 4.0 mg/L + Mel-dep group, SOD activity was lower than in the 4.0 mgCd/L group. A significant positive correlation between SOD activity and Cd concentration in the hepatopancreas was observed after a 3-week depuration period.

##### Discussion

The present study was undertaken to assess the oxidative status of the hepatopancreas during co-exposure to Cd and melatonin in female Prussian carp. For this purpose, the activities of antioxidant enzymes such as GR, GPx, SOD, and the level of GSH were determined in hepatopancreas. Since the oxidative balance of the organism is influenced by Cu, Zn, and Fe, the concentrations of these elements in hepatopancreas were assayed as well.

On the basis of the results obtained in this study, we wanted not only to explain the probable effect of sublethal Cd exposure for 13 weeks on several pollution-indicative biomarker enzymes but also the possible protective effects of melatonin on Prussian carp. To the best of our knowledge, there are no reports on Cd-induced oxidative stress in the hepatopancreas and the role of melatonin in alleviating its toxic effects in Prussian carp. The cadmium had accumulated in the hepatopancreas since the beginning of the exposure, because already after the first week, the level of cadmium increased significantly in all groups exposed to both doses of this metal. Further increases were seen in subsequent weeks of exposure, with the highest level found after 10 weeks. The present findings are consistent with those reported by Al-Asgah et al. (2015), who demonstrated that after long-term exposure to this metal, most cadmium was accumulated in the liver. Studies in a number of fish species demonstrated that the liver is the primary target organ for Cd accumulation and plays an important role in its storage, redistribution, detoxification, and transformation. In the present study, the accumulation of Cd in the hepatopancreas is associated with a significant decrease in hepatosomatic index (HSI) after the 10th and 13th weeks of cadmium exposure. The presented results are similar to the results reported by Van Dyk et al. (2007) and Messaoudi et al. (2009), which investigated the effect of Cd in water on *Oreochromis mossambicus* and *Salaria basilisca* the somatic index of the liver.

The accumulation of Cd in the hepatopancreas may contribute to histopathological changes, such as the loss of normal parenchymal architecture (structure), cytoplasmic vacuolation, cell degeneration, and necrosis (Abalaka 2015; Ghiasi et al. 2017). This damage results from Cd bonding to the sulfhydryl, disulfide, and amine groups of a range of compounds present in the cells, mainly proteins of the cytosol, nucleus, mitochondrial membranes, and lysosomes, which significantly disrupts their homeostasis.

The use of melatonin affected a decrease of Cd concentration in the hepatopancreas of female Prussian carp exposed to the metal. Similar findings have also been reported in mammals (Chwełatiuk et al. 2006; El-Sokkary et al. 2010).

Cadmium is a non-redox metal that may directly induce oxidative stress, causing increased lipid peroxidation, which in turn results in protein modification and membrane gradient alteration, leading to the loss of integrity and irreversible damage (El-Sokkary et al. 2010; Arroyo et al. 2012).

A measurement of liver weight in fish, in relation to hepatic enzyme activities, has been reported in several studies as a useful indicator of exposure to chemical pollutants such as cadmium, as well as of fish health. The liver somatic index is one of the liver measurements that can increase or decrease, depending on the cadmium exposure time. Exposing fish to a sublethal concentration of cadmium usually leads to decreased HSI, as a result of hepatocellular injury associated with cell death (Messaoudi et al. 2009) and possibly due to depletion of energy reserves in liver (Çiftçi et al. 2015). The present study also found a statistically significant decrease of HSI after 10 and 13 weeks of exposure to the highest Cd dose (Table 3). In the present study, Cd and melatonin treatments appeared not to affect HSI levels.

This is supported by the fact that cadmium is able to decrease the HSI and to induce antioxidant enzyme activities in the liver. The liver not only stores xenobiotics, but is also the primary site for detoxification. Fish liver is equipped with an antioxidant defense system. This involves non-enzymatic antioxidants, such as GSH, and enzymatic antioxidants, including SOD, GR, and GPx, all of which protect tissue against the effects of metals (Basha and Rani 2003).

Total antioxidants, including enzyme and non-enzyme antioxidants, play an important role in scavenging cellular reactive oxygen species. According to the present finding, total antioxidant capacity level in the hepatopancreas was significantly decreased in Cd-treated fish (Table 4). Antioxidant depletion following cadmium exposure has also been reported in common carp (*Cyprinus carpio*) (Mehrpak et al. 2015; Banaee et al. 2015). The overproduction of free radicals during exposure to cadmium may be associated with the decrease in the hepatic total antioxidant capacity. Impairment in the synthesis of non-enzymatic and enzymatic antioxidant may be the most important factor in reducing levels of cellular total antioxidant. Therefore, the decline in the hepatic total antioxidant levels make the fish cells more vulnerable to oxidative stress damage. In contrast, administration of melatonin significantly increased the level of total antioxidants in Cd-treated fish (Table 4). The presence of melatonin implants enhanced the total antioxidant capacity of the liver and perhaps protected fish from oxidative stress. Similar results were observed in studies carried on mammals, which documented that increased melatonin levels in the tissues correlate positively with an increase in total antioxidant capacity (Benot et al. 1998, 1999**;** Reiter et al. 2005).

GSH is an important natural antioxidant that prevents free radical damage and contributes to detoxification by conjugating with chemicals. Altered levels of GSH and metabolites, which were found in various studies, indicate that GSH plays a key role in the oxidative-induced toxicity of Cd (Eroglu et al. 2015). In the present study, increased GSH levels were observed in the hepatopancreas of fish exposed to the highest dose of Cd, especially after 4 and 10 weeks of exposure (Table 4). The increase in GSH levels with exposure to Cd was also reported in *Oreochromis niloticus* (El-Gazzar et al. 2014) and in *Channa punctatus* (Dabas et al. 2012). GSH levels in the liver increase due to its enhanced synthesis, increased uptake of amino acid substrates, and activated biosynthesis of enzymes protecting the fish against ROS. Furthermore, GSH can neutralize most of the radicals generated by metal-mediated reactions. Reduced glutathione is believed to act as the first line of cellular protection against metals, because it is the natural chelator for them (El-Gazzar et al. 2014). Moreover, Cd has a high affinity to GSH, which has reactive sites for metals such as cadmium (Jamakala and Rani 2015). In contrast, in the present study, melatonin administration decreased the GSH concentration in groups exposed to cadmium especially after the 4th and 10th weeks of the exposure period. Similar results were observed in the central nervous system and kidney of rats (Ozan et al. 2007; Yildirim et al. 2008).

Glutathione also acts as an important cofactor for antioxidant enzymes such as glutathione reductase (GR) and glutathione peroxidase (GPx) (Mozhdeganloo et al. 2015). The GPx detoxifies H_2_O_2_ derived from oxidative metabolism, as well as peroxides from oxidation of lipids, and is considered the most effective enzyme against lipid peroxidation (El-Gazzar et al. 2014). In the present study, Cd exposure significantly decreased the activity of hepatic GPx after the first, fourth, and seventh weeks of exposure (Table 5). Our results are in accordance with the study performed in fish by Messaoudi et al. (2009) and EL-Gazzar et al. (2014), who indicated that a reduction in GPx activity was noticed when increasing the exposure time to cadmium.

The reduced activities of GPx in the hepatopancreas of fish exposed to cadmium imply heightened free radical injury. The decreased activity of GPx can be interpreted as an indirect effect due to the compromised supply of NADPH and reduced glutathione (GSH), or a direct damage of functional groups (Mukherjee et al. 2010). Furthermore, Cd inhibits the GPx enzyme activity through creates cadmium-selenium complexes in the active center of this enzyme (Sharma et al. 2014). In the present study, melatonin administration in the groups exposed to Cd influenced the increase in the activity of GPx especially after the first, fourth, and seventh weeks of exposure (Table 5). Our results are in agreement with the reported increase in the activities of GPx in brain female and male of rats treated with melatonin (Mukherjee et al. 2010; Shagirtha et al. 2011). Melatonin stimulates the activity of GPx, which reduces free radical damage, because it metabolizes H_2_O_2_ to H_2_O (El-Sokkary et al. 2010). In this process GSH is oxidized to its disulfide, GSSG (glutathione disulfide). GSSG is quickly reduced back to GSH by the enzyme glutathione reductase (GR). In this study, GR activity exhibited little variations compared to other active antioxidant enzymes. Increased GR activity was found after 10 weeks of exposure in the group exposed to the highest dose of Cd (Table 5). Our results are also supported by some other investigators’ findings (Dabas et al. 2012; Eroglu et al. 2015) who worked on cadmium-induced oxidative stress in various fish species. The increased GR activity found in the present study may partially account for the increased glutathione levels in fish exposed to Cd, although the recovery of GSH seems insufficient for alleviating the oxidative stress.

Notably, changes in GPx and GR activity are also associated with changes in the activity of SOD, another antioxidant enzyme comprising the antioxidant defense system of the cells and the body. SOD catalyzes the destruction of O_2_^−^ by dismutation and H_2_O_2_ formation. The results of our study show that the activity of SOD in the hepatopancreas of fish exposed to Cd, especially to 4.0 mg/L, was significantly decreased relative to the other experimental groups after the 1st and 13th weeks (Table 4). A decline in SOD activity in the liver after Cd administration has also been reported for *Rhamdia quelen* (Pretto et al. 2011) and *Oreochromis niloticus* (Saglam et al. 2014). In our studies, administration of melatonin in cadmium-exposed fish showed an increase in the activity of SOD in the hepatopancreas. As reported, melatonin, which is a natural antioxidant, functions directly to scavenge free radicals or indirectly to influence antioxidant enzyme gene expression such as SOD (Rodriquez et al. 2004; Sharma et al. 2014). Our data is in agreement with the study carried out on mammals where a stimulatory effect of melatonin on brain and liver SOD activity was observed (El-Sokkary et al. 2010; Mukherjee et al. 2010; Shagirtha et al. 2011). Nevertheless, the activity of antioxidant enzymes can change depending on the intensity and duration of exposure to cadmium (Pretto et al. 2011). One example of this may be the observed in our research when SOD activity was increased after the 10th week of exposure to cadmium. In the hepatopancreas, the increase in SOD activity was accompanied by the highest accumulation level of cadmium in this tissue. A significant increase in SOD activity in the 10th week of exposure could also be attributed to superoxide radical accumulation. Similar effects were reported in *Channa punctatus* (Dabas et al. 2012) and in *Oreochromis niloticus* (Saglam et al. 2014).

Decreased SOD activity in fish exposed to Cd may be related to the reduced availability of bioelements such as Zn or Cu, which are components of SOD, as a result of their binding to metallothionein. Moreover, SOD activity may also be inhibited by the displacement of Mn ions from the active site of MnSOD and Cu and/or Zn ions from CuZnSOD by cadmium (Jurczuk et al. 2004; Mukherjee et al. 2010; Erdem et al. 2016). It is very probable as an increased accumulation of Zn and Cu was noted in our study of the hepatopancreas in fish exposed to the highest concentration of cadmium (Table 2). Similar results were noted in the study by Jurczuk et al. (2004) and Prozialeck et al. (2016). Administration of melatonin to cadmium-exposed fish influenced a reduction in Zn and Cu concentrations in the hepatopancreas. Melatonin inhibits the increase in Cu and Zn concentrations in the hepatopancreas that could produce oxidative stress.

Apart from copper and zinc, other bioelements analyzed in our investigation are components of different metalloenzymes, such as iron (Fe). Iron, a constituent of catalase, which is an antioxidant enzyme, is essential to life. Several studies have presented that, Fe decomposition in the body tissues is affected by Cd exposure (Jurczuk et al. 2004; Mukherjee et al. 2010). The data obtained in our experiments clearly indicate that Cd exposure affects Fe content in the hepatopancreas of fish exposed to Cd (Table 2). This metal shows biphasic effects (a decrease followed an increase), and Cd could be exerting in variety the effects on uptake and/or iron storage dependent on time exposure. In the present study, the level of Fe in the hepatopancreas of Prussian carp exposed to Cd decreased compared to other groups after a single week of exposure (Table 2). In general, cadmium interferes with the absorption and storage of Fe and low levels of Fe can lead to increased Cd accumulation in tissues such as the liver. Interestingly, from the 7th week of exposure until the end of the study, Fe levels in the hepatopancreas increased in the group exposed to the highest dose of Cd. Our results confirm those of several other studies which have shown that Fe distribution in the tissues is dependent on the time of cadmium exposure and that cadmium can reason redistribution of Fe and exchange of Fe in iron-dependent proteins and enzymes (Djukić-Ćosić et al. 2008; Mukherjee et al. 2010). Melatonin administration also had a two-phase effect. At the beginning of exposure (week 1), melatonin caused an increase in Fe levels in the hepatopancreas in the group exposed to the highest Cd dose but later decreased the Fe levels throughout the exposure period. The simultaneous administration of melatonin restored the previous changes to nearly normal levels.

## Depuration

There are several factors that contribute to the elimination of metal from fish tissues, such as time, fish age, temperature, the metabolic activity of the fish, and the biological half-life of the metal. The excretion of metals from fish body is through the gills, urine, and bile (Kim et al. 2004).

The effectiveness of depuration of cadmium accumulated during 6 weeks’ exposure depended mainly on the duration of the elimination period. Cd elimination from the hepatopancreas in groups previously exposed to Cd was observed after 3 weeks of detoxification. After 6 weeks, there was a slight reaccumulation of Cd in the hepatopancreas in the group previously exposed to the highest doses. Similarly, several previous studies documented that the elimination of accumulated cadmium in the liver of the fish was slow, taking more than 10 days and suggested that Cd, once absorbed into the body, is hardly excreted, but instead is redistributed among tissues (Kim et al. 2004; Thang et al. 2017). In the group with melatonin implant, there was a decrease in cadmium concentration in the hepatopancreas during the depuration phase. This decrease was significantly lower than in the groups without melatonin implant. Melatonin co-treatment during the depuration phase brought about a decrease in the hepatic Cd concentration (Table 3). It is likely that the high concentration of Cd in the hepatopancreas during detoxification decreased HSI in fish previously exposed to the highest Cd dose. In the melatonin-treated groups, HSI remained at levels similar to those found in the controls (Table 3). This is supported by the fact that this metal is able to decrease the HSI and to induce antioxidant enzyme activities in the liver.

An overall increase in the levels of the GSH, FRAP, and GR activity were detected, except for the GPx after 3 weeks’ depuration in the group exposed to the highest dose of cadmium. Interestingly, hepatic GPx activity revealed a significant decrease after third week of depuration, and then increased in sixth week of depuration (Table 5). Ferreira et al. (2007) also found similar changes in GPx activity in the fish liver during the depuration period after exposure to contaminants. During the depuration stage, the hepatopancreas concentrations of heavy metals, i.e., Zn, Cu, and Fe, also changed. An overall increase in the concentrations of these metals was detected after the third and sixth weeks of depuration in the hepatopancreas. The raised concentrations of metals during the 6-week depuration period in the hepatopancreas are possibly the result of repeated accumulation of cadmium released from other tissues during depuration period. The results demonstrated that the evaluation of improvement was faster in the group with melatonin than in the fish left without any exogenous melatonin.

## Conclusion

The present study indicates that exposure to cadmium induces oxidative damage in the hepatopancreas tissue of female Prussian carp which was manifested by a change in the antioxidant defense system. Our findings also showed that administered melatonin is capable of preventing the oxidative toxic effects of cadmium. These studies suggest that melatonin protects the liver of fish from the toxic effects of cadmium on which they may be exposed in the aquatic environment. Hence, it may be useful to use melatonin as a supplement to minimize the toxic side-effects of cadmium.
